# Animal models of heart failure with preserved ejection fraction

**DOI:** 10.1007/s12471-016-0815-9

**Published:** 2016-03-02

**Authors:** G. Conceição, I. Heinonen, A. P. Lourenço, D. J. Duncker, I. Falcão-Pires

**Affiliations:** Department of Physiology and Cardiothoracic Surgery, Faculty of Medicine, University of Porto, Porto, Portugal; Division of Experimental Cardiology, Department of Cardiology, Thoraxcenter, Erasmus MC, University Medical Center Rotterdam, Rotterdam, The Netherlands; Turku PET Centre, University of Turku, Turku, Finland; Department of Anesthesiology, Centro Hospitalar de São João, Porto, Portugal

**Keywords:** Heart failure with preserved ejection fraction, Animal models, Rodents, Diastolic dysfunction

## Abstract

Heart failure with preserved ejection fraction (HFpEF) constitutes a clinical syndrome in which the diagnostic criteria of heart failure are not accompanied by gross disturbances of systolic function, as assessed by ejection fraction. In turn, under most circumstances, diastolic function is impaired. Although it now represents over 50 % of all patients with heart failure, the mechanisms of HFpEF remain understood, precluding effective therapy. Understanding the pathophysiology of HFpEF has been restricted by both limited access to human myocardial biopsies and by the lack of animal models that fully mimic human pathology. Animal models are valuable research tools to clarify subcellular and molecular mechanisms under conditions where the comorbidities and other confounding factors can be precisely controlled. Although most of the heart failure animal models currently available represent heart failure with reduced ejection fraction, several HFpEF animal models have been proposed. However, few of these fulfil all the features present in human disease. In this review we will provide an overview of the currently available models to study HFpEF from rodents to large animals as well as present advantages and disadvantages of these models.

## Introduction

Heart failure is amongst the leading causes of death and disability worldwide and can be defined as the end result of any abnormality in cardiac structure and function that leads to impaired ventricular filling or ejection [1].

Heart failure can be divided into two distinct entities, i.e. heart failure with reduced ejection fraction (HFrEF) and heart failure with preserved ejection fraction (HFpEF). HFrEF is related to systolic dysfunction and characterised by the inability of the myocardium to contract and eject enough blood. In turn, HFpEF is associated with diastolic dysfunction while systolic function is normal or near-normal (hence preserved ejection fraction) [1]. Moreover, HFpEF displays no cardiac dilation, yet is characterised by high filling pressures and lung congestion, dyspnoea and intolerance to effort. Due to these reasons, exercise testing plays an important role in the diagnosis of HFpEF.

**Fig. 1:**
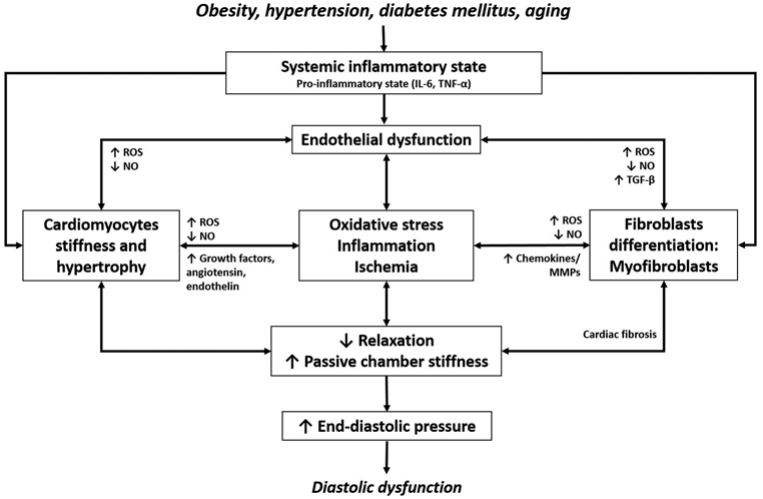
Schematic representation of HFpEF pathophysiology. Comorbidities are associated with systemic changes and myocardial molecular dysfunction translating into structural changes that contribute to HFpEF pathophysiology. *IL-6* interleukin-6, *TNF-α* tumour necrosis factor alpha, *NO* nitric oxide, *ROS* reactive oxygen species, *MMPs* matrix metalloproteinases, *TGF-β* transforming growth factor beta.

**Fig. 2:**
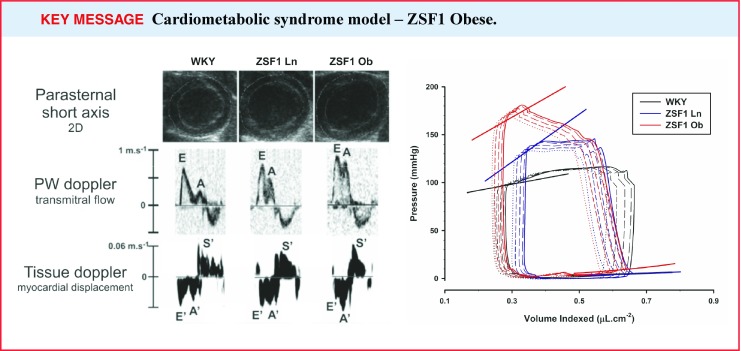
Representative pressure-volume (P-V) loops and echocardiography tracings from Wistar-Kyoto (normotensive control group of obese ZSF1 rats), lean ZSF1 (hypertensive lean control group of obese ZSF1 rats, ZSF1 Ln) and obese ZSF1 (ZSF1 Ob). *A* late diastolic transmitral filling velocities, *A’* late diastolic tissue Doppler mitral annulus velocity, *E* early filling transmitral Doppler velocity, *E’* early diastolic tissue Doppler mitral annulus velocity, *S’* peak systolic tissue Doppler mitral annular velocity

Myocardial remodelling in HFpEF is characterised by abnormal diastolic left ventricular (LV) function resulting from conditions such as increased stiffness, abnormal ventricular-arterial interaction, increased vascular stiffness, endothelial dysfunction, chronotropic incompetence, impaired relaxation and/or cardiovascular reserve function. Among these, the most important mechanisms underlying diastolic dysfunction are disturbed myocardial relaxation and/or altered passive properties of the ventricular wall. Its phenotype is frequently, but not necessarily always, associated with increased interstitial fibrosis with LV concentric remodelling/hypertrophy and atrial enlargement (Fig. 1; [2]). These patients are generally older and mainly female and exhibit a large number of comorbidities, including diabetes mellitus (DM), hypertension, obesity and renal dysfunction [1]. HFpEF is thus an exemplar multifactorial disease and patient heterogeneity is not restricted to the heart, but also involves comorbidities affecting the entire cardiovascular system.

HFpEF is a major and growing epidemiological clinical problem worldwide, whose prevalence has strikingly grown due to increasing prevalence of risk factors in an ageing population [2]. In Europe and in the USA, HFpEF currently accounts for over 50 % of all heart failure cases. Despite being responsible for frequent hospitalisations, high consumption of resources and a prognosis as ominous as that of HFrEF [3], evidence-based therapeutic strategies are still lacking and its treatment remains largely empirical [2]. Indeed, large-scale trials with conventional HFrEF approaches have failed in HFpEF and there is an urgent need to better understand its underlying pathophysiological mechanisms [2]. Thus, so far, treatment options are limited and mostly based on the treatment and relief of other comorbidities.

The development of effective therapeutic strategies for HFpEF is hampered by the lack of appropriate animal models. Only a few animal models of HFpEF have been proposed, reflecting the difficulties in replicating the clinical features of HFpEF in animals. Most of them consist of cardiac pressure-overload models that further develop LV concentric hypertrophy and diastolic dysfunction, but none present all the haemodynamic characteristics of human HFpEF, and few have proven to be reliable for preclinical evaluation of potentially new therapeutic targets. In this review, we present an overview of the currently available animal models that are employed in the study of HFpEF, including rodent and large animal models, and discuss the strengths and weaknesses of these models.

## Animal models of HFpEF

Currently, the limited availability of animal models of HFpEF has potentially represented a major limitation in conducting mechanistic studies and unravelling HFpEF pathophysiology [4]. This is not only due to the absence of clear criteria and cut-off values for the diagnosis of HFpEF in animals but also due to the fact that these experimental models are more challenging, expensive and time consuming when compared to HFrEF. Therefore, the majority of heart failure animal models have been developed for HFrEF. However, animal models of diastolic dysfunction have also become more widely available and closely resemble the pathophysiological mechanisms underlying the disease [5]. The animal models currently available have attempted to reproduce the paramount factors typically documented to cause diastolic dysfunction and HFpEF, namely ageing, diabetes mellitus and hypertension. The animal models highlighted in this review were successfully established in rodents (Table 1), while others were developed in large animals (Table 2).

**Table 1:** Rodent animal models of heart failure with preserved ejection fraction

Ref.	Model	Type	Species	Systolic function	Diastolic function		LV structure	Fibrosis	Features
					Active relaxation	Passive stiffness			
**Aortic Banding and Systemic Hypertension models**									
6	Dahl Salt-Sensitive	G	R	SBP⇧	Tau ⇧	LVEDP ⇧	CH	⇧	Hypertension, IR, dyslipidaemia
8	DOCA-Salt	P/S	R/M	SBP⇧	E/E’ ⇧ E/A ⇩	LVEDP ⇧ Stiffness ⇧	CH	⇧	Hypertension, endothelial dysfunction, oxidative stress, inflammation
10	Hypertrophic agonists	P	R/M	MPI ⇧ LVdP/dt_max_⇩	E/A ⇩ IVRT ⇧ E/E’ ⇧E’ ⇩ Tau ⇧	LVEDP ⇧	CH	⇧	Hypertension
12	Aortic constriction	S	R/M	S ⇧ EF ⇩	E/A ⇧ Tau ⇧ VRT ⇩	LVEDP ⇧	CH	⇧	Hypertension
**Diabetes Mellitus and Obesity models**									
17	*db/db* mice	G	M	E_ES_ ⇩ MPI ⇩	E/A ⇩ LVEDV ⇩	LVEDP ⇧	H	**⬄**	DM type 2, hyperlipidaemia, obesity, IR, hyperinsulinaemia, hyperglicaemia, E_A_ ⇩
18, 19	*ob/ob* mice	G	M	E_ES_ ⇩ CO ⇩	E/A ⇩	Stiffness ⇧	H	**⬄**	DM type 2, leptin deficiency, obesity, hyperglicaemia, hyperinsulinaemia, IR, Apoptosis, E_A_ ⇩
21	Obese Zucker rat	G	R	S ⇩	IVRT ⇧	Stiffness ⇧	H	⇧	Obesity, hyperphagia
22	ZDF	G	R	Intact	E/A ⇩	Stiffness ⇧	H	**⬄**	Obesity, hyperinsulinaemia, hyperglycaemia, hyperleptinaemia
24	OLETF	G	R	S ⇩	DT ⇧ E/A ⇩	Stiffness ⇧	H	⇧	Hypertension, DM type 2
**Cardiometabolic syndrome models**									
25	Dahl/SS/Obese	G	R	ND	E/A ⇩ Tau ⇧	LVEDP ⇧	H	⇧	Obesity, hypertension, dyslipidaemia, IR, DM type 2, oxidative stress, inflammation
26, 27	ZSF1 obese	G	R	Preserved	Tau ⇧ E/E’ ⇧ Restrictive LV inflow signal	LVEDP ⇧ Chamber and myocardial stiffness ⇧	CH	**⬄**	Obesity, DM type 2, IR, hyperinsulinaemia, hypertiglycedaemia, hypercholesterolaemia, hypertension
**Ageing models**									
29	FVB/N mice	G	M	⇩	E/A ⇩	Time to peak diastolic filling⇧	CH	⇧	Age-dependent diastolic dysfunction in male mice
28	SAMP8	G	M	=	E/A ⇩ E’ ⇩ E’/A’ ⇩	LVEDP ⇧ Chamber and myocardial stiffness ⇧	**⬄**	⇧	–
30, 31	Fischer 344	G	R	EF ⇩	IVRT ⇧ E’ ⇩	Stiffness ⇧	H	⇧	Female display more differences

*ND* Not determined, *LVdP/dt*_*max*_ Maximum rate of rise of left ventricular pressure, *E*_*ES*_ End-systolic elastance, *E*_*A*_ arterial elastance, *Tau* time constant of relaxation, *LVEDP* LV end-diastolic pressure, *DM* diabetes mellitus, *S* peak systolic velocity, *E/A* ratio between early (E) and late (A) diastolic transmitral filling velocities, *SBP* systolic blood pressure, *DT* deceleration time, *E’* early diastolic tissue Doppler mitral annulus velocity, *IVRT* isovolumetric relaxation time, *G* genetic, *S* surgical, *P* pharmacological, *MPI* myocardial performance index, *IR* insulin resistance, *CO* cardiac output, *EF* ejection fraction, *R* rat, *M* mouse, *EF* ejection fraction, *CO* cardiac output, *H* hypertrophy, *CH* concentric hypertrophy

**Table 2:** Large animal models of heart failure with preserved ejection fraction

Ref.	Species	Disease model	Systolic function	Diastolic function		LV structure	Fibrosis	Arterial stiffness	Coronary vascular dysfunction
				Active relaxation	Passive stiffness				
41	MC	Renal wrap	ND	ND	ND	ND	Progressive ⇧ over 80 wk	ND	ND
40	D	Renal wrap	LVdP/dt_max_⇧	Tau ⇧	Chamber and myocardial stiffness ⇧	CH	ND	ND	MBF ⬄
40	D	Renal wrap	E_ES_ ⇧	Tau ⇧	LVEDP ⇧ Stiffness ND	CH	ND	ND	ND
37	D	Renal wrap	E_ES_ ⇧	Tau ⇧	LVEDP ⇧ Stiffness **⬄**	CH	⇧	E_A_ ⇧	ND
39	D	Renal wrap in young + old animals	E_ES_ ⇧	Tau ⇧	**⬄**	CH	⇧	E_A_ ⇧	ND
39	D	Renal wrap in old animals	E_ES_ ⇧	**⬄**	E_ES_ ⇧ Fpass ⇧	–	⇧	E_A_ ⇧	ND
36	Sn	Aortic banding	Contractile reserve ⇩	Tau ⇧	ND	H	⇧	ND	ET-1 responses ⇧ Ca^2+^ signalling ⇩
35	Sn	Gradual aortic cuff inflation	**⬄**	Tau ⇧	Stiffness ⇧	H	⇧	ND	ND
42	MR	Spontaneous DM type 2	S ⬄ or somewhat ⇩	Variable, E’ and E/A ⇩ or pseudonormal	ND	A trend to thinner walls in diabetes	ND	ND	ND

*ND* Not determined, *LVdP/dt*_*max*_ maximum rate of rise of left ventricular pressure, *E*_*ES*_ end-systolic elastance, *Tau* time constant of relaxation, *MBF* myocardial blood flow, *LVEDP* LV end-diastolic pressure, *E*_*A*_ arterial elastance, *F*_*PASS*_ cardiomyocyte passive force, *ET-1* endothelin-1, *DM* diabetes mellitus, *S* peak systolic velocity, *E’* early diastolic tissue Doppler mitral annulus velocity, *E/A* ratio between early (E) and late (A) diastolic transmitral filling velocities, *D* dog, *Sn* swine, *MR* macaque rhesus, *MC* macaque cynomolgus, *CH* concentric hypertrophy, *H* hypertrophy

### Rodent animal models of HFpEF

#### Aortic banding and systemic hypertension

Hypertension represents a major risk factor for several heart diseases and an important contributor to HFpEF [4], showing a prevalence of 55–86 % in HFpEF patients [4]. Thus, not surprisingly, many models of HFpEF have hitherto involved studies of increased afterload and LV concentric hypertrophy (i.e. transverse-aortic constriction or systemic arterial hypertension) [4].

The Dahl salt-sensitive (SS) rat was selectively bred from Sprague-Dawley presenting the highest values of blood pressure. Dahl/SS is characterised by hypersensitivity to sodium intake and represents the most published HFpEF animal model [6]. When fed with a high-salt diet (8 % NaCl) from the age of 7 weeks, Dahl/SS rats develop renal failure, fast-developing hypertension (> 175 mmHg) and LV hypertrophy, falling into HFpEF between 12 and 19 weeks of age [6]. At week 12, Dahl/SS rats develop diastolic dysfunction such as increased chamber stiffness (left and upward shift of end-diastolic pressure-volume relationship (EDPVR) and decrease of end-diastolic volume). Between 16 and 20 weeks, Dahl/SS hearts enlarge so that end-diastolic volumes and EDPVRs become similar to the respective age-matched controls. Concomitantly, overall pump function curves move towards control, and ejection fraction declines, thus, progressing towards HFrEF. Despite normal or enhanced overall pump function at a certain time-point, LV end-diastolic pressure (LVEDP) and wet lung weight increases, indicating the development of heart failure. Caution should be taken as some authors report HFrEF rather than HFpEF in Dahl/SS. It appears that subtle differences in the experimental design, such as the specific genetic background of the animals as well as environmental conditions, including diet, may importantly impact the cardiovascular phenotype of Dahl/SS.

The deoxycorticosterone acetate (DOCA) salt-induced rat model represents a pharmacologically induced model of hypertension. DOCA is administered by intraperitoneal or subcutaneous injection at 7 weeks of age, 1 week after a unilateral nephrectomy. This combination results in hypertension, renal hypertrophy, nephrosclerosis, cardiac hypertrophy and perivascular fibrosis within 4–5 weeks of chronic DOCA treatment [7]. It is used in rats and mice, and isotonic saline is the sole drinking fluid, which accelerates and aggravates hypertension progression [8]. The DOCA-salt hypertensive rat develops myocardial inflammation, oxidative stress, fibrosis and diastolic dysfunction. Diastolic dysfunction was confirmed by impaired relaxation (higher E/E’ and time constant of isovolumetric relaxation - τ) and increased stiffness (elevated EDPVR slope) compared to control subjects [8] and this seems to be related with the impact of oxidative stress on myofilamentary proteins [8]. Subsequently, a related model was developed, consisting of exposure to 2 weeks of transverse-aortic constriction prior to DOCA administration, demonstrating normal LV systolic pressure and fractional shortening but more hypertrophy, fibrosis and diastolic dysfunction (higher LVEDP and EDPVR) with increased lung weight, consistent with HFpEF [9].

Chronic stimulation with pro-hypertrophic agents, such as angiotensin II and isoprenaline, has been used as a model of systolic and diastolic dysfunction as well as LV hypertrophy. Angiotensin II-treated rodents show evidence of hypertension, increased LV hypertrophy, fibrosis and expression of natriuretic peptides, which result in diastolic dysfunction confirmed by increase/worsening in LV isovolumetric relaxation time (IVRT), myocardial performance index, τ, LVEDP, E/E’ along with decreased E/A, without changes in LV dimensions, mass, or ejection fraction [10]. In the same way, the use of isoprenaline displayed cardiac hypertrophy, myocardial fibrosis and decreased ventricular relaxation [11]. Notwithstanding, blockers of the renin-angiotensin-aldosterone system or β-adrenergic receptors have not demonstrated appreciable benefit in patients with HFpEF.

Transverse-aortic constriction is a well-established surgical technique for inducing LV chronic pressure overload and hypertrophy in rodents. Moderate transverse-aortic constriction imposed at an early age triggers concentric LV hypertrophy with compensated chamber performance, markedly with prominent diastolic filling abnormalities. Transmitral flow profile showed a ‘restrictive’ filling pattern with increased early (E-wave) and decreased late (A-wave) diastolic transmitral filling velocities, augmented E/E’ and left atrial diameter, more rapid deceleration of the early filling wave as well as a shortened IVRT. These abnormalities became progressively more exaggerated at 12 and 18 weeks [12], thus representing a good model for studying HFpEF. The transition from compensated hypertrophy to early failure (HFrEF) is heralded by LV dilation, impairment of systolic function and progression of the abnormal filling [12].

A major limitation of using aortic-banded or hypertensive models is that the majority of patients with HFpEF continue to have heart failure symptoms even when the blood pressure is controlled. Conversely, less than 50 % of HFpEF patients have LV hypertrophy and often show no evidence of LV dilatation, thus making the value of the current preclinical model questionable [13].

#### Diabetes mellitus

Approximately one-third of HFpEF patients have type 2 DM and cardiovascular disease is the leading cause of morbidity and mortality in diabetic patients. Interestingly, diastolic dysfunction represents an early cardiac manifestation of the deleterious effects of DM, since young diabetic individuals predominantly display diastolic abnormalities, whereas HFrEF rarely develops before middle age in obese diabetic individuals [14]. Insulin resistance, type 2 DM and hyperinsulinaemia exert a number of pleiotropic effects on the myocardium, including stimulation of hypertrophy, increased oxidative stress and proinflammatory/profibrotic effects, which can induce deleterious changes in cardiomyocyte function as well as in the extracellular matrix [15].

Many models of type 2 DM recapitulate the features of HFpEF patients, including leptin-deficient *ob/ob* mice and the leptin receptor-deficient *db/db* mice, whose altered leptin homeostasis leads to obesity due to hyperphagia, hyperglycaemia, hyperinsulinaemia, insulin resistance and diabetic complications at different time points [16].

*Db/db* mice develop cardiac hypertrophy as evidenced by increased LV mass and wall thickness at 9 weeks of age, which is associated with a smaller LV end-diastolic volume. The progression of diabetic cardiomyopathy is accompanied by increased production of reactive oxygen species and interstitial fibrosis [17]. Five-month-old mice display haemodynamic alterations such as elevated LVEDP and EDPVR, reduced dP/dt_min_, and prolonged relaxation. At this age, transthoracic echocardiography confirmed preserved systolic function with elevated IVRT and reduced E/A, clearly indicating diastolic dysfunction [17]. The administration of angiotensin II for 4 weeks in *db/db* mice induced the expression of markers of hypertrophy and fibrosis but did not impact greatly on cardiac structure or lead to HFpEF [18].

*Ob/ob* mouse is an obesity and diabetic animal model that shows evidence of diastolic dysfunction possibly due to cardiac lipid accumulation [19]. *Ob/ob* mice develop myocardial hypertrophy and triglyceride accumulation, which parallels LV diastolic dysfunction as confirmed by decreased E/A [19]. Progressively, this animal model develops diabetic cardiomyopathy with impaired contractility and relaxation [20].

#### Obesity

The increase in global incidence of obesity predicts a continuous rise of the burden of cardiovascular diseases. This is particularly true for HFpEF, as obesity has a prevalence of 41–46 %, is associated with an increased risk of hypertension, dyslipidaemia and diabetes and thus represents an independent risk factor for its development [21]. Many available models of obesity are derived from selective crossing between rats comprising one out of the two most significant mutations in leptin receptor (i.e. *fa* and *cp*). These models are usually associated with other risk factors, including hypertension and/or diabetes, with some of them developing diastolic dysfunction or HFpEF.

Zucker rats were originally bred to be a genetic model of obesity and hypertension. The obese Zucker rat demonstrates a consistent increase in LV mass and early diastolic dysfunction with prolonged IVRT [22]. In contrast, the obese diabetic Zucker (ZDF) rat does not develop increased LV mass even though mild hypertension is present and diastolic dysfunction is evident [23].

The Otsuka Long-Evans Tokushima Fatty (OLETF) rat was derived by selecting spontaneously diabetic rats displaying polyuria, polydipsia and obesity from an outbred colony of Long-Evans rats [24]. The OLETF rat is used as a model of diabetic cardiomyopathy and displays echocardiographic evidence of diastolic dysfunction from 20 weeks of age as observed by deceleration time of the E-wave and decreased E/A [25].

#### Cardiometabolic syndrome

Dahl/SS/obese rats, derived from Dahl/SS and Zucker crossing, have been recently established as a new model of metabolic syndrome. At 15 weeks of age, female Dahl/SS/obese rats present with LV diastolic dysfunction, marked LV hypertrophy and fibrosis, associated with increased cardiac oxidative stress and inflammation [26].

A recently discovered HFpEF model is the obese ZSF1 rat. The ZSF1 rat was generated by crossing non-hypertensive lean female Zucker diabetic fatty rats (ZDF, +/*fa*) with lean spontaneously hypertensive heart failure prone male rats (SHHF/Mcc, +/*facp*), sharing a common genetic background with Wistar Kyoto rats [27]. Both lean and obese ZSF1 animals inherit a hypertensive gene from the spontaneously hypertensive rat strain and show elevated blood pressure [27]. The 20-week-old obese ZSF1 rat represents a robust model of metabolic syndrome since it displays hypertension, obesity, type 2 DM, insulin resistance, hyperinsulinaemia, hypertriglyceridaemia, hypercholesterolaemia and heart failure. Considering such features, this cardiometabolic risk model develops diastolic dysfunction such as prolonged τ, an upwards shift of EDPVR and elevated arterial elastance. Along with higher resting LVEDP, obese ZSF1 rats show reduced effort tolerance and VO_2_max, showing, for the first time in an animal model, an important corresponding feature of HFpEF human diagnosis - exercise intolerance. In parallel, there is LV hypertrophy and left atrial dilation (Fig. 2), without signs of renal failure at 20 weeks of age [28]. In male obese ZSF1 rats, the increased myocardial stiffness appears mostly due to myofilament changes, without significant interstitial fibrosis [28]. A subgroup of ZSF1 obese rats subjected to a Western high-fat diet for 10 weeks showed no further aggravation of HFpEF phenotype [28].

#### Ageing

The prevalence of HFpEF in the community increases with advancing age and is higher in women. Indeed, the reported age- and sex-specific prevalence for individuals 80 years and older is about 4–6 % in men and 8–10 % in women [21]. Thus, ageing models are important to understand the pathophysiology of age-related diastolic dysfunction.

The spontaneous senescence-prone (SAMP8) mouse offers a good model of cardiac dysfunction associated with ageing. At 6 months of age, this animal model displayed a significant reduction of E/A ratio, E’ and E’/A’ along with increased EDPVR, fibrosis and TGF-β expression when compared to the control group (SAMR1). No differences in systolic function or mean arterial pressure were reported in SAMP8 mice [29].

FVB/N mice represent a robust inbred strain and males display diastolic dysfunction at 12 months, with an E/A < 1 while, in females, this phenotype is not observed [30]. In contrast, Fischer 344 ageing rats showed more prominent LV hypertrophy and diastolic dysfunction in females compared to males, as assessed by increased IVRT and decreased septal E’. Instead, males displayed fibrosis and systolic dysfunction [31]. Other studies aiming to evaluate age-related cardiac changes using male Fischer 344/Brown Norway F1 rats revealed a decline in cardiac function and LV structural changes, compatible with the phenotype of HFpEF [32].

### Large animal models of HFpEF

Although the large number of rodent models has importantly contributed to our understanding of the underlying causes and mechanisms of LV diastolic dysfunction, rodents have inherent limitations due to their size, cardiac structure and function compared to larger mammalian species, in particular the human heart. Therefore, experimental models of human heart failure have also been recapitulated in large animal models, which have been particularly useful in elucidating several important pathophysiological aspects of diastolic dysfunction and HFpEF (Table 2; [5]).

#### Aortic banding

A considerable number of aortic banding studies have been performed and many have yielded significant insights into the pathophysiology of HFpEF, well before the actual term was even established. The most consistent finding from pressure-overload studies is the principally concentric type of LV hypertrophy that is produced and which is often observed in HFpEF. In addition, these studies [33–35] have revealed the presence of impaired myocardial blood flow reserve, particularly during exercise, impaired endothelial function of large coronary artery, enhanced apoptosis, abnormalities in bioenergetics and metabolism, exhausted myocardial perfusion and exaggerated oxygen consumption during exercise, all of which are likely contributors to diastolic dysfunction in this model [36]. Nonetheless, cardiac dysfunction is often variable, as in patients, and not always even evident as assessed in vivo although abnormalities at the myocyte level are still present [37]. Furthermore, these earlier studies concentrated mostly on functional aspects and, apart from increased fibrosis [38], histological information from these studies remains limited. Increased LV fibrosis was documented by Yarborough et al. who also reported increased LV diastolic stiffness in a model of HFpEF induced by progressive aortic cuff inflation [39]. In their study, increased LV fibrosis was due to enhanced collagen stability but not its expression, and collagen content correlated with myocardial stiffness [39]. Aortic banding-induced HFpEF was recently investigated by Emter and colleagues [33, 38]. In support of earlier findings, these authors documented enhanced fibrosis in the left ventricle of aortic banded swine, but also showed that exercise training is a promising treatment in HFpEF, as it promotes compliant extracellular matrix fibrotic components and preserves extracellular matrix regulatory mechanisms. In addition, exercise training preserved myocardial oxygen balance, and promoted physiological molecular hypertrophic signalling cardiac phenotype [33, 38]. This is important, as no proven pharmacological treatments have yet been reported for HFpEF.

#### Renal wrapping

Renal wrapping-induced hypertension has been extensively used in dogs. Hart and colleagues reported that this model increased LV fibrosis and mass, which was accompanied by elevations in LVEDP 12 weeks after renal wrapping [40]. In their study, LV end-diastolic volume, ejection fraction, stroke volume, cardiac output and neurohumoral activity including circulating angiotensin II, endothelin and catecholamine levels as well as plasma renin activity were all comparable with control levels [40]. In contrast, humoral activation (renin activity, aldosterone, brain natriuretic peptide and adrenomedullin) was increased in bilateral renal wrapping performed by Maniu and colleagues [41]. To our knowledge, the first large animal study designed to specifically address HFpEF was performed by Munagala et al. [42], who showed that bilateral renal wrapping-induced hypertension resulted in increased arterial stiffness, associated with an increased LV mass and fibrosis in old dogs, although LV volume and also LV diastolic stiffness were similar to healthy controls [42]. These authors also reported enhanced end-systolic elastance in this model of HFpEF. Renal wrapping in dogs has also been studied by Hayashida and colleagues, for testing of novel pharmacological treatments for HFpEF [43], and this model continues to be used. Renal wrapping was also applied in an early study in nonhuman primates by Abrahams and colleagues, who observed a progressive increase in LV collagen content, with cardiomyocytes ultimately becoming encased by collagen after 80 weeks of follow-up [44].

#### Obesity, metabolic syndrome and diabetes mellitus animal models

In comparison with studies in rodent models (Table 1), obesity or metabolic syndrome (such as high fat diet alone or in combination with experimental diabetes) induced HFpEF has been sparsely studied in large animal models. Diastolic dysfunction, albeit in a variable degree, was however documented recently in type 2 DM rhesus monkeys (Table 2), although LV histology was performed in only one diseased animal [45]. Furthermore, several large animal models with features of metabolic syndrome have demonstrated alterations in coronary vascular function and structure. For instance, Trask et al. described in obese Ossabaw swine with metabolic syndrome an hypertrophic inward remodelling of coronary resistance microvessels along with coronary capillary rarefaction, which was associated with a decrease in coronary flow and myocardial ischaemia [46]. In a pre-atherosclerotic diabetes mellitus porcine model with type 2 characteristics small coronary arteries displayed reduced nitric oxide bioavailability and loss of endothelin-1 response [47]. Unfortunately, cardiac function was not investigated, and thus future studies are required to assess the impact of these coronary vascular abnormalities on cardiac function.

#### Ageing models

With advancing age, the cardiovascular system is known to stiffen, while the prevalence of HFpEF increases. Hence, ageing dogs have been used to investigate the effect of age on features of HFpEF. Ageing alone did not have a major influence on LV structure and function in the study by Munagala et al., but the combination of old age and renal wrapping resulted in impairments in diastolic function although myocardial fibrosis was similar to control levels [42], suggesting that hypertension rather than age was the causal factor [42]. Conversely, a decrease in LV distensibility was observed in aged compared to young dogs, which was accompanied by an increase in LVEDP and a decrease in LV end-diastolic volume [48]. As ejection fraction and LV mass were not affected, this model recapitulates several aspects of HFpEF and may prove useful in future studies.

## Discussion

The use of animal models has proven to be an extremely valuable tool in understanding the pathophysiology of complex cardiovascular diseases such as HFpEF, even considering that the experimental results are not readily transferable to human patients. In this regard, rodents present distinct cardiac features, such as its structure and higher heart rates, which often preclude obtaining reliable isovolumetric relaxation times and mitral valve velocities, especially when non-invasive techniques, such as echocardiography, are used to determine cardiac function. In contrast, large animal heart failure pathophysiology is more similar to that of humans, but ethical concerns and difficulty in achieving trans-genesis in large animals still limit research. Not surprisingly, heart failure models were originally developed in rodents because of the numerous advantages inherent to a small animal model, including lower housing and maintenance costs, thus allowing a larger number of animals to be included in a study and improving its statistical power. Moreover, recent technological advances in echocardiography, MRI, and micromanometer conductance catheters have greatly facilitated assessment of cardiac function in rodents, removing a significant barrier to their use in heart failure research [49]. Among rodents, the major advantage of mice compared to rats is the fact that pharmacological studies become less expensive as the drug is usually administered proportionately to body weight. Mice major limitation is the amount of cardiac mass to perform post-mortem analysis. With regard to diastolic dysfunction, it should be emphasised that rodent models generally progress to HFrEF within a variable amount of time, which means that, in those animals, HFpEF is merely a temporary step in the development of HFrEF. In contrast, several human pathologies are characterised by stable and isolated HFpEF, which does not necessarily progress towards HFrEF.

A general limitation of most animal models of heart failure is the sudden onset of heart failure due to a surgical or drug intervention, whereas human heart failure generally develops over a period of several years. Moreover, in humans, HFpEF is a condition typically associated with ageing and the diastolic dysfunction/HFpEF animal models are typically relatively young. Finally, human HFpEF is often associated with comorbidities, including atherosclerosis, hypertension, diabetes or obesity, while development of atherosclerosis is rather rare in most rodent strains, at least without genetic modifications [50].

## Conclusion

Several animal models of HFpEF have been developed that recapitulate some, but not necessarily all, of the characteristics described in HFpEF patients. This significantly limits their usefulness in developing novel therapeutic strategies for HFpEF. Researchers need to decide what risk factor or combination of risk factors leading to this multifactorial disease should be included in their protocol design according to the purpose of study. The choice of appropriate models is thus essential and will lead to better understanding and finding of new knowledge in this increasingly prevalent disease.

### Funding

This work was supported by the Portuguese Foundation for Science and Technology (PTDC/BIM-MEC/0998/2012, partially funded by FEDER), through the Cardiovascular R&D Unit (FCT nº. 51/94) and the Academy of Finland, Finnish Diabetes Research Foundation, and Finnish Foundation for Cardiovascular Research.
